# Molecular analysis of *Dirofilaria repens* removed from a subcutaneous nodule in a Japanese woman after a tour to Europe

**DOI:** 10.1051/parasite/2015002

**Published:** 2015-01-27

**Authors:** Jun Suzuki, Seiki Kobayashi, Utako Okata, Hitomi Matsuzaki, Mariko Mori, Ko-Ron Chen, Satoshi Iwata

**Affiliations:** 1 Division of Clinical Microbiology, Department of Microbiology, Tokyo Metropolitan Institute of Public Health 3-24-1 Hyakunincho Shinjuku-ku, Tokyo 169-0073 Japan; 2 Department of Infectious Diseases, Keio University School of Medicine 35 Shinanomachi Shinjuku-ku, Tokyo 160-8582 Japan; 3 Department of Dermatology, Saiseikai Central Hospital 1-4-17 Mita Minato-ku, Tokyo 108-0073 Japan; 4 Department of Dermatology, Keio University School of Medicine 35 Shinanomachi Shinjuku-ku, Tokyo 160-8582 Japan

**Keywords:** *Dirofilaria repens*, Imported dirofilariasis, Ribosomal RNA genes, Mitochondrial genes, Phylogenetic analysis

## Abstract

A premature female *Dirofilaria* species, subsequently identified as *Dirofilaria repens* by its morphological features and mitochondrial 12S ribosomal RNA (*12S rRNA*) gene sequence, was removed from a subcutaneous nodule of the right temporal region of the head in a Japanese woman 2 years after she noticed swelling of her left calf following an insect sting during a tour to Europe; headache symptoms were noticed a few months later. The sequences of the mitochondrial *12S rRNA* and cytochrome c oxidase subunit I genes from the organism were almost identical to those of sequences AM779772 (100% homology, 337/337) and AM749233 (99.8% homology, 536/537) of *D. repens* isolated from humans in Italy. However, the phylogenetic position of the *18S rRNA*-internal transcribed spacer 1-5.8S rRNA region was in the same cluster as that of sequence JX290195 of *Dirofilaria* sp. “*hongkongensis*” (96.7% homology, 348/360), which was recently reported from Hong Kong as a novel *Dirofilaria* species. Information on regional genetic variation in *D. repens* isolated from animals and humans remains scarce. We report the detailed genetic features of this filaria as a reference isolate from a specific endemic area, to enrich the genetic database of *D. repens*.

## Abstract (fr)

Une femelle immature de *Dirofilaria*, par la suite identifiée comme *Dirofilaria repens* par ses caractéristiques morphologiques et la séquence du gène de son ARN ribosomique mitochondrial 12S (ARNr 12S), a été retirée d’un nodule sous-cutané de la région temporale droite de la tête d’une femme japonaise, deux ans après qu’elle ait remarqué un gonflement de son mollet gauche suite à une piqûre d’insecte lors d’un voyage d’agrément en Europe. Des symptômes de maux de tête ont été remarqués quelques mois plus tard. Les séquences des gènes de l’ARNr mitochondrial 12S et de la sous-unité I de la cytochrome c oxydase de l’organisme étaient presque identiques à celles des séquences AM779772 (100 % d’homologie, 337/337) et AM749233 (99,8 % d’homologie, 536/537) de *D. repens,* isolées chez l’homme en Italie. Cependant, la position phylogénétique de la région intercalaire 1-5.8S de l’ARNr 18S était dans le même groupe que celui de la séquence JX290195 de *Dirofilaria* sp. “*hongkongensis*” (96.7 % d’homologie, 348/360), qui a été récemment rapporté à Hong Kong comme une nouvelle espèce de *Dirofilaria*. Les informations sur la variation génétique régionale de *D. repens* isolés chez les animaux et les humains restent rares. Nous rapportons les caractéristiques génétiques détaillées de cette filaire comme isolat de référence d’une zone endémique spécifique, pour enrichir la base de données génétique de *D. repens*.

## Introduction

*Dirofilaria repens* Railliet & Henry, 1911 [21] infects dogs, cats, and other carnivores in the Old World. However, in Japan, *D. repens* is an uncommon parasite (no cases of infection with *D. repens* in domestic dogs have been reported as of 2014), and in the majority of animal and human dirofilariasis cases, *Dirofilaria immitis* was identified as the etiological agent. However, although the sources of infection are not clear, two human cases caused by domestic infection of *D. repens* have been reported in Japan [11, 12].

Here we report a suspected case of imported dirofilariasis in a Japanese woman, caused by *D. repens* from Europe. Dirofilariasis caused by *D. repens* is highly prevalent in the Mediterranean region of Southern Europe (e.g., Spain, the south of France, and Italy) [17]. In Italy, 298 human cases have been reported, and in Bulgaria, there have been an increasing number of people infected by *D. repens* in recent years [10]. Moreover, mosquitoes that were positive for *D. repens* were found in northern Germany in 2011 and 2012 [3]. However, information on the regional genetic variation of *D. repens* is still scarce. In addition, as a novel *Dirofilaria* species, *Dirofilaria* sp. “*hongkongensis*” has been reported from Hong Kong [25], based on the sequence homology of the 18S-internal transcribed spacer 1 (ITS1)-5.8S rRNA region, a reference for the differentiation of filarial species [13].

Creation of a complete genetic database of every *Dirofilaria* species, including *D. repens*, from specific endemic areas is essential for the correct differentiation of *Dirofilaria* species, and enrichment of this database will be valuable to facilitate the diagnosis, proper treatment, and prevention of vector-borne diseases such as dirofilariasis following a trip abroad. In the present study, we analyzed the features of samples from *Dirofilaria* species (a fresh female *Dirofilaria* specimen in the present case, and the present female and male *D. immitis* isolates preserved in 70% ethanol). Thus, we report the detailed genetic information of the *12S rRNA*, *COI*, and *18S rRNA* genes and sequences of the *ITS1* region of these two species to enrich the genetic database of *Dirofilaria*.

## Materials and methods

### *Dirofilaria* species and *D. immitis* isolates

The live, premature adult female *Dirofilaria* isolate, subsequently identified as *D*. *repens* by its morphological features and mitochondrial *12S rRNA* gene sequence, was removed from a subcutaneous nodule on the right temporal region of the head in a Japanese woman (approximately 40 years of age) 2 years following the appearance of swelling of her left calf and headache symptoms a few months after returning from a tour of European countries (“Romantische Straße” of Germany, Belgium, The Netherlands, and Sardinia island in Italy) for 16 days in August, 2012. The swelling appeared shortly after an insect sting on Sardinia island. The large central portion of the present *Dirofilaria* isolate was fixed with 70% ethanol and prepared for paraffin embedding.

The female and male adults of *D. immitis* from a Japanese dog were preserved in 70% ethanol and kindly provided by the Tokyo Metropolitan Animal Care and Consultation Center.

### Paraffin embedding

The cross-sections were processed for paraffin embedding by using a graded series of ethanol, xylene, and paraffin according to the conventional method. Small pieces (5–6 mm in length) were cut from 70% ethanol-fixed specimens and placed upright by positioning them between slices of the thigh muscles of a frog specimen preserved in 70% ethanol.

### Scanning electron microscope (SEM) observations

The cut portions from the central part of adult females of the *Dirofilaria* isolate and the *D. immitis* isolate preserved in 70% ethanol were fixed with 2.5% glutaraldehyde/phosphate buffer, pH 7.2, for 1 h. The specimens were immersed in t-butyl alcohol after dehydration in a graded series of ethanol (50–100%), attached on the specimen stub with double-sided adhesive carbon tape, and frozen for 40 s in liquid nitrogen. The frozen samples were immediately mounted on the specimen stage of the SEM (JSM-5600LV; JEOL Ltd.; Akishima, Tokyo, Japan) and slowly sublimated for 30 min. The freeze-dried samples were coated with Pt-Pd by using an ion sputter, and the samples were then remounted on the specimen stage of the SEM and observed at an accelerating voltage of 4 kV.

### Polymerase chain reaction (PCR) and sequence analysis

The DNA was extracted using a QIAamp DNA Mini Kit (Qiagen, Venlo, The Netherlands) from approximately 50 mg of the *Dirofilaria* isolate and of each of the *D. immitis* specimens. PCR amplification of each DNA template was performed using primer sets targeting the *12S rRNA* (Diro12S-F/Diro12S-R primer set based on GQ292761), *18S rRNA* (Diro18S-F1/Diro18S-R1 and Diro18S-F2/Diro18S-R2 primer sets based on AF036638), and *COI* (Diro-cox1-F/Diro-cox1-R primer set based on AF271614 and NC_005305) genes, and the *ITS1* (Diro18S-F3/Diro5.8S-R1 primer set based on AF217800) region in the genus *Dirofilaria* (Table 1). PCR was performed in a reaction mixture (50 μL) containing 2 μL of DNA template, 1.0 U ExTaq DNA polymerase (Takara Bio Inc.; Shiga, Japan), 0.4 μM of each primer, and 0.25 mM of deoxynucleotide triphosphates. The following cycling parameters were used for all PCR amplifications: (1) Taq activation at 94 °C for 5 min; (2) 35 cycles of denaturation at 94 °C for 30 s, annealing at 60 °C (*18S rRNA*, *ITS1*, and *12S rRNA*) or 54 °C (*COI*) for 30 s, and extension at 72 °C for 1 min; and (3) final extension at 72 °C for 5 min. The amplified *ITS1* fragment was cloned using the Mighty TA-cloning Kit (Takara Bio Inc.; Shiga, Japan). The PCR products were sequenced using the ABI Prism BigDye Terminator v3.1 Cycle Sequencing Ready Reaction Kit and an ABI PRISM 3500 Genetic Analyzer (Applied Biosystems Japan Ltd.; Tokyo, Japan).

**Table 1.** Oligonucleotide primers used for PCR assays in the present study.

Primer name	Primer sequence (5′–3′)	Position	Accession no.
Diro12S-F (forward)	CATTTTAATTTTTAACTCTATTT	22–44	GQ292761
Diro12S-R (reverse)	GATGGTTTGTACCACTTTAT	483–502	GQ292761
Diro18S-F (forward)	CCATGCATGTCTAAGTTCAA	2–21	AF036638
Diro18S-R (reverse)	TCGCTACGGTCCAAGAATTT	872–891	AF036638
Diro18S-F2 (forward)	CTGAATACTCGTGCATGGAA	755–744	AF036638
Diro18S-R2 (reverse)	TTACGACTTTTGCCCGGTT	1706–1724	AF036638
Diro18S-F3 (forward)	AATTCCTAGTAAGTGTGAGTCATC	1533–1556	AF036638
Diro5.8S-R (reverse)	TAGCTGCGTTCTTCATCGAT	540–559	AF217800
Diro-cox1-F (forward)	GCTTTGTCTTTTTGGTTTACTTTT	1–24	AF271614
Diro-cox1-R (reverse)	TCAAACCTCCAATAGTAAAAAGAA	1067–1090	NC_005305

### Phylogenetic analyses

Multiple alignments and phylogenetic analyses of the obtained sequences of the *12S rRNA* and *COI* genes and the *ITS1* region of the two *Dirofilaria* species were performed using Clustal W [24] and the maximum likelihood (ML) (PHYML version 2.4.5 software [8]) and Bayesian (MrBayes version 3.1.2) methods [22]. The ML method and a general time-reversible (GTR) model were used to calculate genetic distances. Statistical support was evaluated using bootstrapping of 1000 replicates for the ML method. In the Bayesian analysis, we ran four simultaneous chains (nchain = 4) for 1,000,000 generations with an initial burn-in of 1250, at which point the likelihood values had stabilized. The GTR model with a proportion of invariant bases and four categories of among-site rate variation were used, and trees were sampled every 100 generations. The ML tree and Bayesian tree data files were visualized using MEGA version 4.0.2 [23]. The GenBank accession numbers and strain names of the reference *Dirofilaria* species used in these phylogenetic analyses are shown in Figure 2. New sequences were deposited in GenBank (accession numbers AB973225–AB973231).

## Results

### Morphological features

**Figure 1.**
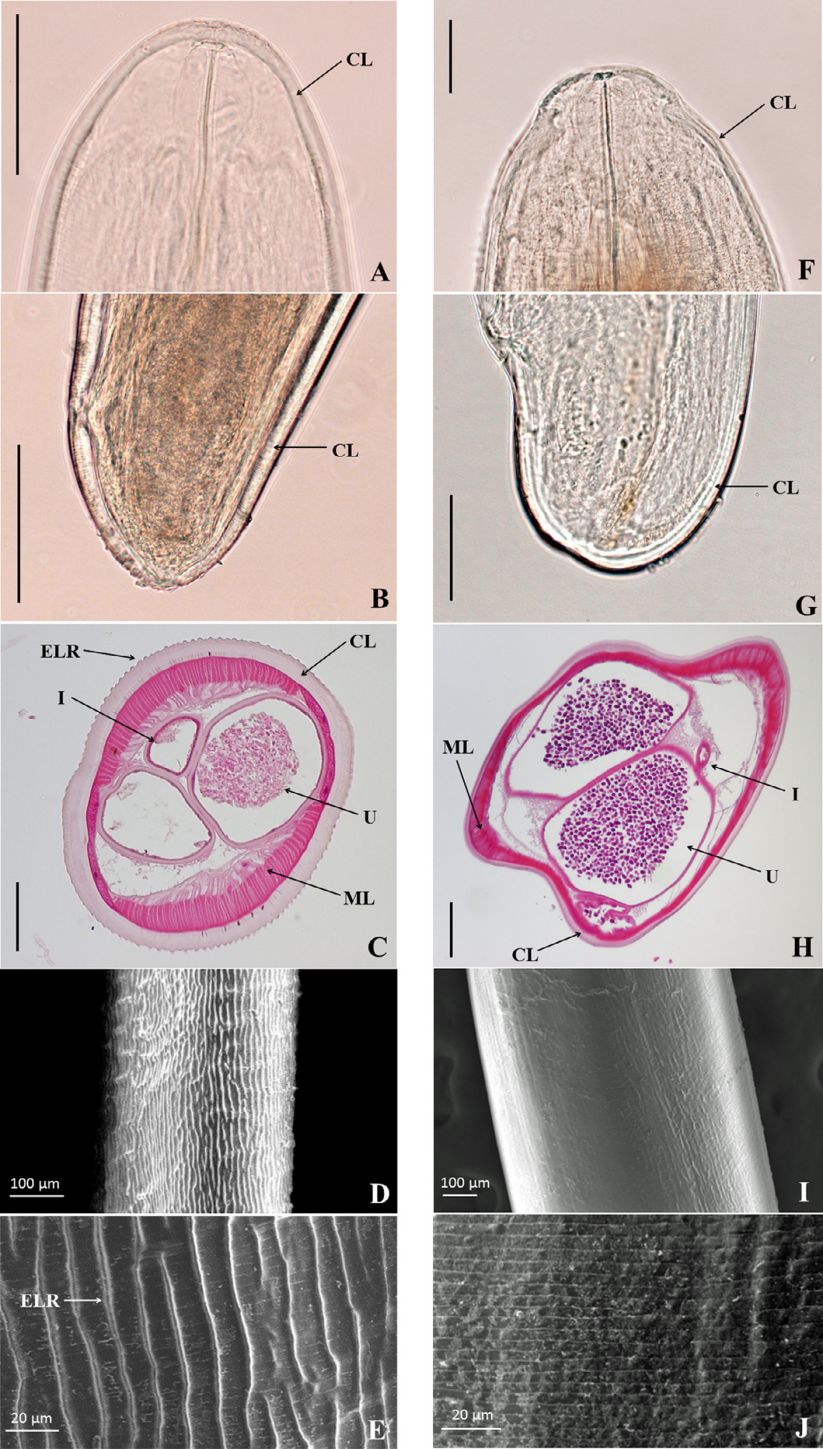
Morphological images of female adults of the *Dirofilaria* species isolated from the present case (left column, A–E) and *Dirofilaria immitis* (right column, F–J). A, F: direct images of the cephalic parts under an optical microscope. B, G: direct images of the caudal parts under an optical microscope. C, H: cross-sectional tissue sections (hematoxylin and eosin stain). D, I: low-magnification images of the body surfaces under a scanning electron microscope (SEM). E, J: high-magnification images of the body surfaces under SEM. Scales bars: A, B, C, F, G, 100 μm; H, 200 μm. CL, Cuticular Layer; ELR, External Longitudinal Ridge; I, Intestine; ML, Muscular Layer; U, Uterus.

The premature adult *Dirofilaria* female isolated in this case (119 mm in length and approximately 460 μm in diameter) had been continually moving in saline for several hours after surgical removal from the patient. The surface of the *Dirofilaria* isolate had a pattern indented by clear external longitudinal ridges (Figs. 1C–1E), similar to that of premature and mature *D. repens* removed from human patients [5, 7, 9, 16] (Table 2). In contrast, the adult female of *D. immitis* (289 mm in length and approximately 1020 μm in diameter) did not show a clearly defined ridged body pattern (Figs. 1H–1J). The pattern of ridges of the *Dirofilaria* species isolated from the present case differed from the crested longitudinal ridges (~5-μm interval) of *Dirofilaria ursi* from a human patient [27]. The measured values of the external longitudinal ridges of the *Dirofilaria* species in the present case were 3–4 μm in height, and they were spaced at 15–17 μm intervals and numbered 118–122. These values are generally consistent with values reported for adult female *D. repens* removed from three human patients (Table 2), and their morphologies differ from those of *D. immitis* [19], *Dirofilaria tenuis* [15], and *D. ursi* [2, 27, 28]. The curved line on the top of the head of the present *Dirofilaria* species showed a more smoothed, obtuse angle accompanied by a continuous thick cuticular layer (Fig. 1A) than that of the *D. immitis* female (Fig. 1F). Conversely, the curved line of the caudal end of this *Dirofilaria* species showed a more acute angle (Fig. 1B) than that of the *D. immitis* specimen (Fig. 1G). The thickness of the cuticle layer of the *Dirofilaria* species (Fig. 1C: 27–36 μm) was greater than that of the *D. immitis* specimen (Fig. 1H: 11–22 μm). The number of somatic muscles per quadrant was 15 (Table 2).

**Table 2.** Morphological aspects of female *Dirofilaria repens* in infected humans.

References	Present study	Gardiner et al. (1978) [7]	Gutierrez et al. (1995) [9]	Otranto et al. (2011) [16]	Elsayad et al. (2012) [5]
Length (mm)	119	–	–	117	120 and 110
Breadth (mm)	0.46	0.55	0.22–0.66	017–0.53	0.34 and 0.29
Number of somatic muscles per quadrant	15	15	–	–	–
Thickness of cuticular layer (μm)	27–36	16–25	–	–	16–48
External longitudinal ridge					
Height (μm)	3–4	3–4	–	–	4
Interval (μm)	15–17	15–20	12	7–12	13
Number	118–122	–	95–105	–	–

### Molecular identification and phylogenetic analysis

**Figure 2.**
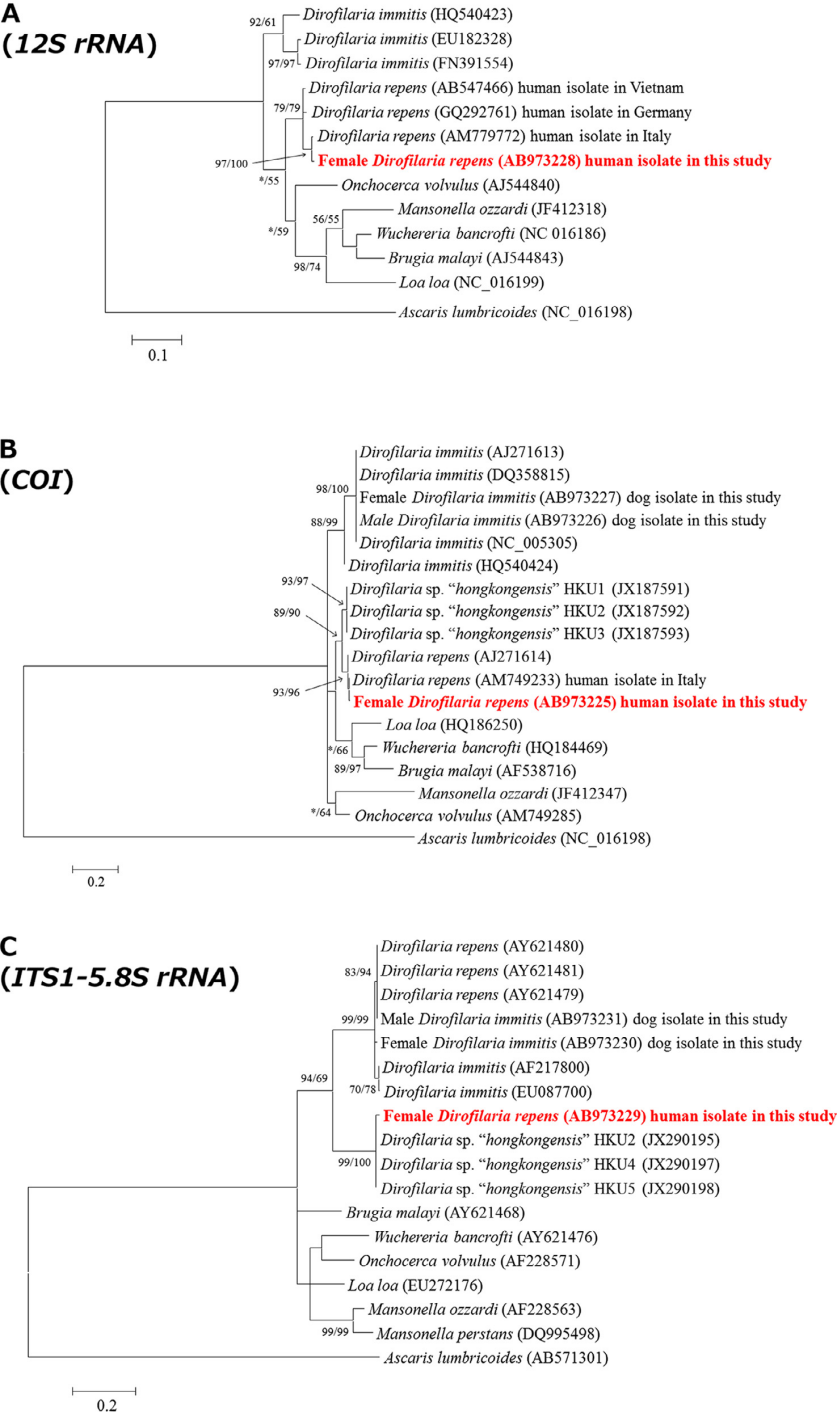
Phylogenetic relationships by maximum likelihood (ML) analysis among sequences of mitochondrial 12S ribosomal RNA (A), mitochondrial cytochrome c oxidase subunit 1 (B) genes, and the internal transcribed spacer 1-5.8S ribosomal RNA region (C). The ML tree was derived from a general time-reversible model using a discrete gamma distribution (+G) with five rate categories and invariant sites (+I). Significant bootstrap support for the ML analysis with 1000 replicates and Bayesian analysis (BI) are shown above the nodes in the order ML/BI. An asterisk indicates <50% support for a node. The scale bar represents the genetic distance in single nucleotide substitutions. GenBank accession numbers are given within parentheses.

The 337 bp sequence of the *12S rRNA* gene of the present *Dirofilaria* species (AB973228) was 100% identical with that of *D. repens* (AM779772) [6] isolated from a human in Italy, and was 98.5% similar (5 bp differences in 338 bp) to that of *D. repens* (AB547466) [4] isolated from a human in Vietnam. The sequence of the *COI* gene of the present *Dirofilaria* species (AB973225) was 99.8% identical (1 bp difference in 537 bp) to that of strain AM749233 [6] of *D. repens* isolated from a human in Italy (Table 3). The phylogenetic positions of the *12S rRNA* and *COI* genes of the *Dirofilaria* species were also classified into the same cluster as AM779772 [6] and AM749233 [6] of *D. repens* (Figs. 2A and 2B).

**Table 3.** Similarities (%) in the *COI* gene sequences among *Dirofilaria repens* and *Dirofilaria immitis*.

	*Dirofilaria repens* in this study (AB973225)	*Dirofilaria repens* (AJ271614)	*Dirofilaria repens* (AM749231)	*Dirofilaria* sp. “*hongkongensis*” (JX187591)	*Dirofilaria immitis* in this study (AB973227)
*Dirofilaria repens* (AB973225)	–	99.3% (566/570)	99.8% (536/537)	95.3% (306/321)	89.9% (425/473)
*Dirofilaria repens* (AJ271614)	99.3% (566/570)	–	99.8% (560/561)	96.0% (308/321)	90.3% (427/473)
*Dirofilaria repens* (AM749231)	99.8% (536/537)	99.8% (560/561)	–	95.6% (307/321)	90.1% (426/473)
*Dirofilaria* sp. “*hongkongensis*” (JX187591)	95.3% (306/321)	96.0% (308/321)	95.6% (307/321)	–	89.1% (286/321)
*Dirofilaria immitis* (AB973227)	89.9% (425/473)	90.1% (426/473)	90.1% (426/473)	89.1% (286/321)	–

Parentheses under scientific name: GenBank accession numbers.

However, the sequence of the *ITS1* region of the present *Dirofilaria* species was classified into the same cluster with strains JX290195 [25] of *Dirofilaria* sp. “*hongkongensis*” (96.7% homology (348/360) identity with JX290195 from a human). In addition, the sequences (AB973230 and AB973231) of the female and male adults of *D. immitis*, respectively, isolated from a dog and analyzed in this study were classified near the cluster of *D. repens* (AY621480, AY621481, and AY621479) rather than that of *D. immitis* (AF217800 and EU087700) (Fig. 2C).

Since no information on *18S rRNA* gene sequences of *D. repens* are registered in GenBank, the sequence of the *18S rRNA* gene of the present *Dirofilaria* species (AB973229) was compared with that of the adult female *D. immitis* isolate (AB973230) from a dog and with a reference isolate (AF182647) [26] of *D. immitis* from a dog. The variation of the sequence between the present *Dirofilaria* species (AB973229) and the present *D. immitis* (AB973230) or a reference *D. immitis* (AF182647) was 96.6% similar (60 bp differences in 1760 bp) and 96.4% similar (48 bp differences in 1332 bp), respectively.

### Identification of the present *Dirofilaria* species

The sampled *Dirofilaria* species was considered to be *D. repens* based on its morphological features and sequence identities of mitochondrial *12S rRNA* and *COI* genes, although the sequence of the *ITS1* region was different from those of *D. repens* reference strains reported in GenBank (Fig. 2C), as observed by phylogenetic analysis.

## Discussion

The endemic area of *D. repens* is widespread in the Old World (Eurasia and sub-Saharan Africa), and regional genetic diversity of *D. repens* has been described for the *12S rRNA* [6, 20] gene, the *COI* [6] gene, and the *ITS1* region [13, 25] and reported in GenBank. However, detailed information of the regional genetic variation in the prevalence of *D. repens* is still unclear owing to various factors – isolates can only be obtained surgically; samples are fixed with formalin; detailed genetic analysis is costly; etc. In Japan, the total number of cases of dirofilariasis in humans was on the rise up to 2002, which was similar to the trend observed in Bulgaria [10], and pulmonary and extra-pulmonary dirofilariasis has accounted for 254 and 26 cases, respectively, since 1964 [1]. *D. immitis* was reported to be the causative agent in almost all these cases and was mostly diagnosed morphologically. However, according to a serological epidemiological study conducted in districts in Tokyo, the recent prevalence of *D. immitis* among shelter dogs has decreased in recent years [14]. Up until 2014, only three cases of domestic dirofilariasis caused by *D. repens* have been reported in Japan, including the present case, and all *D. repens* parasites have been isolated from humans. The present case was strongly suspected to be one of imported dirofilariasis caused by *D. repens* because the sequence of the *12S rRNA* gene of the present *Dirofilaria* species (AB973228) was 100% homologous to that of *D. repens* (AM779772) [6] isolated from a human in Italy and because the patient was stung by an insect in Sardinia island, Italy, which is an endemic area of human dirofilariasis caused by *D. repens* [18].

Misidentification of *Dirofilaria* species is likely among cases diagnosed only by morphology owing to the difficulties in the identification in the immature stage of *Dirofilaria* parasites and because of poor sampling conditions, as described in a review on *Dirofilaria* species isolates that were reported as *D. immitis* [19]. In Japan, some cases of subcutaneous human dirofilariasis that are similar to that caused by *D. repens* have been diagnosed as *D. immitis* infections based on morphological or serological analysis without molecular identification. In the present study, identification of *Dirofilaria* species through both morphological and genetic analyses proved to be extremely helpful.

In our study, a sequence of the *ITS1* region of the sampled *Dirofilaria* species was classified into the same cluster as isolates of *Dirofilaria* sp. “*hongkongensis*”, whereas those of the present *D. immitis* specimens were classified close to the cluster containing *D. repens* isolates. These results suggest the existence of polymorphic variation in the *ITS1* sequence in *Dirofilaria* species. However, the genetic database of *Dirofilaria* species is not yet sufficient to fully evaluate this possibility; therefore, further enrichment of a detailed species-specific genetic database will be required.
